# The European green toad, *Bufotes viridis*, in Donaufeld (Vienna, Austria): status and size of the population

**DOI:** 10.3897/herpetozoa.34.e75578

**Published:** 2021-11-18

**Authors:** Amir Sistani, Stephan Burgstaller, Günter Gollmann, Lukas Landler

**Affiliations:** 1 University of Vienna, Department of Evolutionary Biology, Djerassiplatz 1, 1030 Vienna, Austria; 2 Institute of Zoology, University of Natural Resources and Life Sciences, Gregor-Mendel-Straße 33/1, 1180 Vienna, Austria

**Keywords:** Amphibia, Bufonidae, conservation, population ecology, urban environment

## Abstract

The European green toad, *Bufotes viridis* (Laurenti, 1768), is a rare and protected species in Vienna. In spring and summer 2020, we conducted a survey to assess size and status of its population in Donaufeld, an agricultural area designated for real estate development. Recaptures of photographically registered toads allowed to estimate the population size with 137 individuals (confidence interval: 104–181). Comparatively large body size indicates the presence of a well-established population. Reproductive success was high in the study year. A mismatch mating of a male *B*. *viridis* with a female *Bufo bufo* was observed. Mitigation measures are needed to support this population facing imminent habitat deterioration.

## Introduction

The European green toad, *Bufotes viridis*, was described by Josephus Nicolaus Laurenti from Vienna, where he found it in dark clefts and hollows of the city’s walls: “Habitat inter fissuras, seu cavernas murorum obscuras Viennæ” (Laurenti 1768). Ever since then, this species has survived in the expanding city, constantly shifting its distribution. Green toads have been characterized as steppe dwellers, adapted to dry open habitats with low gappy plant cover. In Central Europe *B*. *viridis* is regarded as a synanthropic species, occurring mainly in agricultural landscapes with a warm climate (Stöck et al. 2009). It also lives in gardens, parks and ruderal areas in urban environments (Kovács and Sas 2010; Kaczmarski et al. 2019; Konowalik et al. 2019).

In Vienna most of the previously occupied sites were no longer inhabited by the 1980s (Cabela 1990). This decline was attributed to the obliteration of summer habitats, intensification of road traffic, destruction of breeding sites, and application of biocides in agriculture (Cabela 1990). Green toads lived up to their reputation as a pioneer species, rapidly colonizing newly created water bodies such as the Marchfeldkanal, an irrigation channel constructed for improving ground water supply in an arable region east of Vienna (Cabela and Girolla 1994), as well as ponds and bays on the Danube Island (Cabela et al. 2003). Owing to succession, most of these places were quickly abandoned by the toads. A survey of the northern and southern outskirts of Vienna yielded a number of new breeding sites in 2010 (Csarmann et al. 2010); several of which were no longer occupied seven years later (pers. obs.). More recently, *B*. *viridis* turned up in quarters closer to the city center, such as a park in a housing estate built at the site of a former railway station (Csarmann 2012), or commercial gardening areas in Simmeringer Haide (Staufer 2018).

*Bufotes viridis* was also sighted in Donaufeld, an agricultural area that has been assigned to real estate development. Until 2016, no amphibian species had been recorded from there in the Austrian Herpetofaunistic Data Base (HFDÖ; Schweiger et al. 2016). Despite its high protection status – *B*. *viridis* is included in Annex IV of the Habitats Directive and designated a priority species in Vienna’s Nature Conservation Ordinance – the species was largely neglected in pertinent planning processes. This imminent threat prompted us to initiate a survey of the population, to assess its size and status. In the course of this investigation, we unexpectedly also observed the presence of another toad species, *Bufo bufo* (Linnaeus, 1758).

## Methods

### Study Area

**Figure 1:**
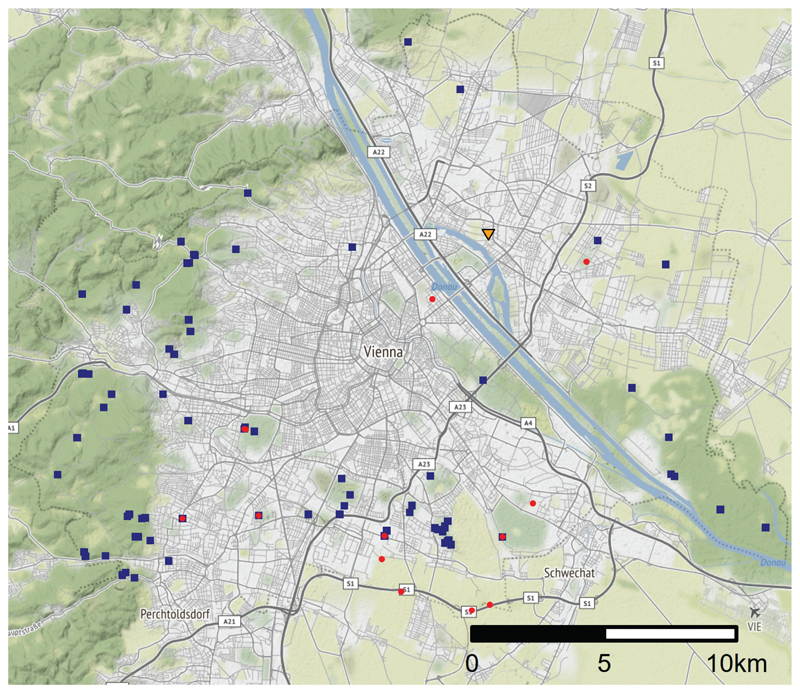
Map of Vienna showing the location of the breeding site in Donaufeld (triangle) and breeding sites of *B*. *viridis* (red circles) and *B*. *bufo* (blue squares) recorded in 2015 and 2016 (Schweiger et al. 2016). Source: OpenStreetMap contributors (2020) Planet dump [Data file from 20 September 2021].

Donaufeld is located north of Alte Donau, a former branch of the river Danube which was converted into a recreational lake in the course of river regulation in the late 19th century. At that time, the floodplain forest was cleared and the area developed for commercial gardening. It is a flat, open area of about 66 ha, which in the study year presented a rural appearance, consisting of a mosaic of farms growing vegetables and fruit, allotment garden areas, fallows, and arable fields (many of the gardeners had sold their properties to real estate developers). This area is isolated by large expanses of built-up areas and major roads from the nearest previously known breeding sites of both *B*. *viridis* and *B*. *bufo* (Fig. 1).

Donaufeld has no natural water bodies. There were a few small ponds on private property, of which only one could be surveyed regularly. Most observations of green toads were made in a shallow sealed depression located beside the road called “An der Schanze” (48.2484°N, 16.4255°E), which was filled with water from an irrigation pipe in late April. Henceforth, we refer to this puddle as “the breeding site”.

### Field Work

The area was surveyed from 22 April to 19 July 2020 two or three times a week during the evening hours and at night, resulting in 30 visits. The following data were recorded from all captured toads: snout-vent length (SVL), body mass, sex and the exact location of the individual. To keep the toads in temporarily safe custody, several plastic buckets were used. Snout-vent length was measured with vernier-calipers to the nearest 0.1 mm. Body mass was weighed with a digital micro-scale (Model: Hoosiwee Präzision Taschenwaage, 1000 g) to the nearest 0.1 g. For weighing the toads, a plastic measuring cup was placed on the scale. Sex was determined by morphological and behavioural features, in particular the nuptial pads of the front legs, the distinct mating call of the males as well as the male exclusive release call. Toads below 50 mm SVL without male characters were classified as juveniles. Photographs of the dorsal pattern of the toads were taken in a plastic box with in-glued shrink-wrapped millimeter paper. In addition, the presence of tadpoles and metamorphs was recorded at the breeding site, but no formal attempts at estimating their numbers were undertaken.

### Individual Identification

To support individual identification of toads from the photographs, the program IBEIS was used (Crall 2020). This is a follow-up version of the software HotSpotter (Crall et al. 2013), which performed best in a pilot study with photos of green toads (Burgstaller et al. 2021). For these comparisons, the head region from the snout to the front back, including the parotid glands, was determined as region of interest. All pictures were also checked visually, to avoid overlooking any identification.

### Data analysis

All analyses and data plots were made in R (R Core Team 2020). The median for body mass and SVL for each individual was calculated and then screened for outliers using the *boxplot* function in R, identified outliers were then removed from the data set before plotting and analyses of SVL and mass. Weather data were obtained from the “Zentralanstalt für Meteorologie und Geodynamik” from weather station Donaufeld (48.2572°N, 16.4314°E). For plotting these data, a 3-day average for temperature and daily summed rain was used.

For demographic analysis we used the function *open-CR*.fit (package openCR (Efford 2020)) and estimated the population size using the Jolly-Seber-Schwarz-Arnason model for open populations (type ‘JSSAb’ in openCR). The full model included two time variables (continuous and non-continuous time effects) for the parameters apparent survival, entry and detection probability. We performed AIC-based model selection of all combinations. The model with the lowest AIC and without any possible maximization errors was then used to estimate the population size (Burnham and Anderson 2002).

## Results

**Figure 2:**
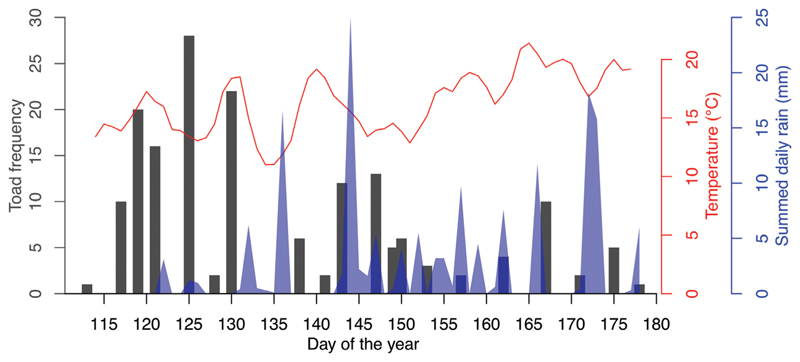
Captures of *B*. *viridis* (Toad frequency = number of captures) and weather conditions during the sampling period.

A total of 170 capture events of green toads were recorded, from which 79 individuals were identified. Of the 79 individuals, 61 were males, 15 females and 3 juveniles. Most individuals were captured only once, but a few were frequently recaptured, up to 9 times (Table 1). Most captures were made in late April and May, the last adult was captured on 26 June 2020 (Fig. 2). Capture frequency showed no clear relation to weather conditions.

**Figure 3:**
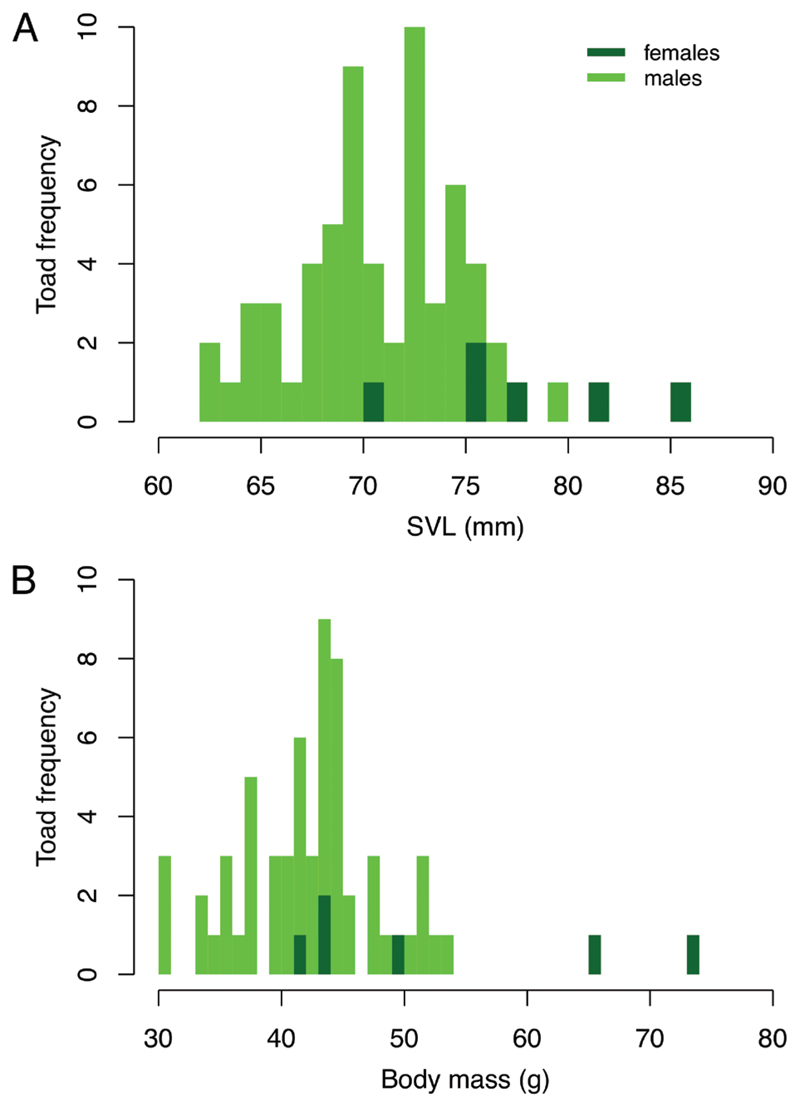
Distribution of snout-vent length and body mass of adult *B*. *viridis* (Toad frequency = number of individuals; for toads captured more than once, median values of the measurements were used).

Average snout-vent length of the males was 70.8 mm (SD: 3.81 mm), females were on average larger with a SVL of 78.2 mm (SD: 5.2 mm) (Fig. 3). Average SVL of the juveniles was 43.8 mm (SD: 3.4 mm). Mean body mass of the males was 42.0 g (SD: 5.57 g), the value for females was 52.0 g (SD: 13.3 g). The juveniles weighed on average 10.1 g (SD: 1.4 g).

The best demographic model (AICc = 961.9) included non-continuous time for capture probability, however, all other parameters were not time dependent (see supplementary file 1: Table S1). The population size was estimated with 137 (confidence interval: 104–181) individuals. Capture probability was highest towards the beginning of the monitoring effort and decreased in the second half of the sampling period. The second-best model had already a considerably higher AICc (973.4), therefore, no model averaging was considered.

From 5 May to the middle of June 2020, large numbers of tadpoles were observed at the breeding site. The first metamorph was recorded there on 15 June. Metamorphosis of hundreds of toadlets continued during the following weeks.

**Figure 4:**
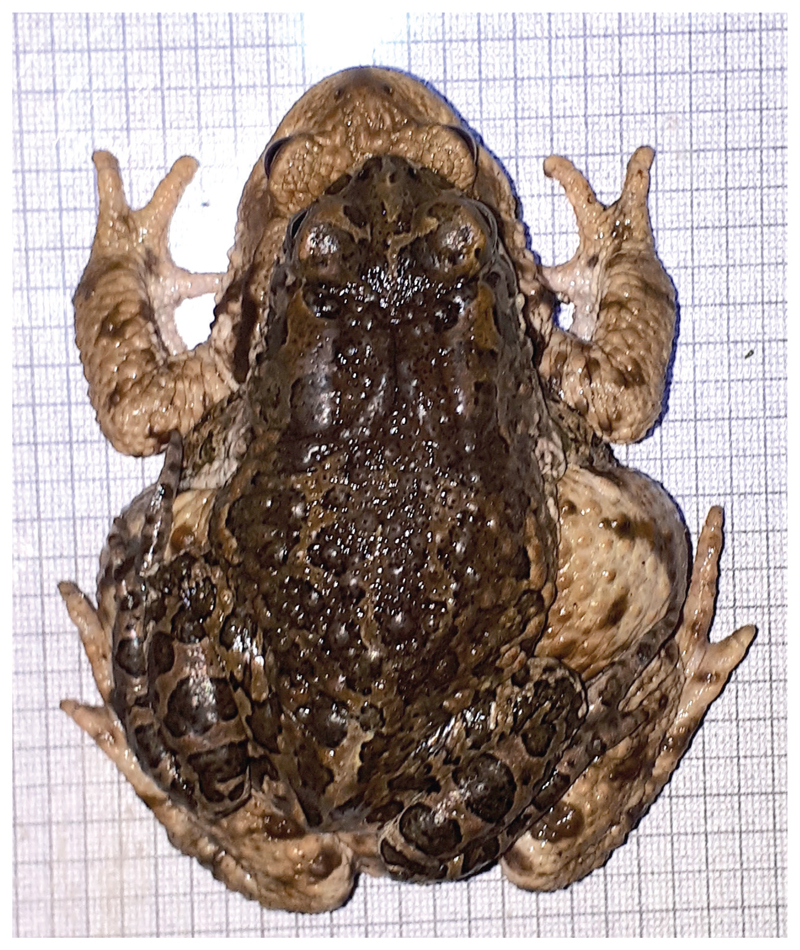
Amplexus of male *B*. *viridis* with female *B*. *bufo* (30 April 2020).

Besides green toads, also common toads, *B*. *bufo*, were encountered in Donaufeld. On 28 April a single male was observed on the road approximately 200 m west of the breeding site, close to a blackberry farm. On 30 April a pair of common toads in amplexus was found at the breeding site, as well as a mismatch amplexus of a green toad male with a common toad female (Fig. 4).

## Discussion

Our survey confirmed the presence of a *B*. *viridis* population in Donaufeld whose size was larger than expected based upon the scanty information previously available. If we consider the whole study area, and not only the breeding site, the size of the adult population is probably underestimated by the recapture analysis. The strongly male-biased sex ratio in our sample is typical for studies centered on water bodies where the males spend more time than the females who just visit for mating and spawning (Kovács and Sas 2010; Kaczmarski et al. 2019). Observed activity showed no clear relation to weather conditions, presumably because water supply for the main spawning site was largely independent of precipitation. Reproductive success was high in the year 2020, albeit at a single breeding site.

Size structure indicates the presence of a well-established population. Snout-vent length in *B*. *viridis* is related to age, and to a minor extent also to habitat quality (Sinsch et al. 2007). Green toads in Donaufeld were larger and heavier than those studied in Rudolf-Bednar-Park, another recently discovered population in central Vienna. There SVL averaged 64.9 mm for males and 68.8 mm for females in 2016, whereas mean body mass was 29.8 g for males and 37.3 g for females (Wappl and Heyer 2016). This difference may be caused by age structure, due to higher survival and perhaps less regular breeding success in Donaufeld, by better growing conditions in Donaufeld, or by a combination of both.

Generally, the two toad species appear spatially segregated in Central Europe, with *B*. *bufo* living in woods and breeding in large permanent ponds, and *B*. *viridis* inhabiting open areas and spawning in shallow, temporary water bodies. In Warsaw, spatial separation of the two species increased over time, with *B*. *viridis* surviving closer to the city center, and *B*. *bufo* at its periphery (Mazgajska and Mazgajski 2020). Also in Rome, green toads – now assigned to *Bufotes balearicus* (Böttger, 1880) – did not breed in ponds where *B*. *bufo* was present (Ensabella et al. 2003). Different choice of habitats and the ability of *B*. *bufo* to tolerate lower temperatures and the presence of fish may explain this pattern, whereas the role of competition remained unstudied (Ensabella et al. 2003). In a laboratory experiment with animals collected north of Vienna, tadpoles of *B*. *viridis* were superior competitors in relation to *B*. *bufo* (Katzmann et al. 2003).

In extensive garden areas, both species apparently find suitable terrestrial habitats. Low population densities and scarcity of water bodies may promote their syntopic occurrence and mismatching matings (Zavadil and Roth 1997; Duda 2008). Most offspring of these interspecific matings die in early developmental stages (Hertwig et al. 1959; Canestrelli et al. 2017), but occasionally some survive to adulthood. An adult male hybrid was found in a garden pond in Perchtoldsdorf, south of Vienna (Duda 2008). Further investigations of interactions between the two species in Donaufeld should be worthwhile.

## Conclusion

Our results demonstrated the presence of a reproductively active population of *B*. *viridis* in Donaufeld. Subsequently, conservation authorities decided that a new pond has to be constructed before the existing breeding site may be destroyed in building activities. This measure may save the population in the near future. If, eventually, much of the open areas disappear in the course of housing development, the chances for survival of the green toads there will severely decrease.

## Supplementary Material

Supporting Information

## Figures and Tables

**Table 1:** Capture frequencies of *B*. *viridis* in Donaufeld.

	Number of captures									
	1	2	3	4	5	6	7	8	9	10
Males	27	14	7	4	5	1	1	0	1	1
Females	13	2	0	0	0	0	0	0	0	0
Juveniles	2	1	0	0	0	0	0	0	0	0
