# Supplementary material for: The European green toad, Bufotes viridis, in Donaufeld (Vienna, Austria): status and size of the population
Source: Herpetozoa. Author manuscript; Available in PMC 2022 Apr 18. (PMC7612631; doi:10.3897/herpetozoa.34.e75578)
Supplement: Supporting Information [file EMS144263-supplement-Supporting_Information.pdf]

Table S1. The ten best population models from model selection. Shown are the model formula, ΔAICc and the estimate for the population size (Super N).

Model terms	ΔAICc	Super N estimate (lower CI - upper CI)
p~t; phi~1; b~1; superN~1	0.00	137 (104 - 181)
p~Session; phi~1; b~Session; superN~1	11.45	111 (87 - 143)
p~1; phi~Session; b~Session; superN~1	24.17	113 (88 - 144)
p~Session; phi~ Session; b~Session; superN~1	26.96	108 (81 - 145)
p~Session; phi~1; b~1; superN~1	28.82	176 (135 - 229)
p~1; phi~Session; b~1; superN~1	37.71	119 (93 - 153)
p~Session; phi~Session; b~1; superN~1	38.45	198 (154 - 253)
p~t; phi~1; b~Session; superN~1	38.93	112 (83 - 149)
p~Session; phi~Session + t; b~Session; superN~1	100.12	109 (81 - 145)
p~Session; phi~ Session + t; b~1; superN~1	108.09	197 (154 - 253)

Names follow the convention from the openCR package: p = capture probability, phi = apparent survival, b = entry probability, SuperN = superpopulation size, t = non-continuous time effect; Session = continuous time effect.
